# Morphological Characters of the Thickbody Skate *Amblyraja frerichsi* (Krefft 1968) (Rajiformes: Rajidae), with Notes on Its Biology

**DOI:** 10.1371/journal.pone.0039963

**Published:** 2012-06-29

**Authors:** Carlos Bustamante, Julio Lamilla, Francisco Concha, David A. Ebert, Michael B. Bennett

**Affiliations:** 1 School of Biomedical Sciences, The University of Queensland, St Lucia, Queensland, Australia; 2 Laboratorio de Elasmobranquios (ELASMOLAB), Instituto de Ciencias Marinas y Limnológicas, Universidad Austral de Chile, Valdivia, Chile; 3 Laboratorio de Biología y Conservación de Condrictios, Facultad de Ciencias del Mar y de Recursos Naturales, Universidad de Valparaíso, Chile, Reñaca, Viña del Mar, Chile; 4 Pacific Shark Research Center, Moss Landing Marine Laboratories, Moss Landing, California, United States of America; 5 Research Associate, South African Institute for Aquatic Biodiversity, Grahamstown, South Africa; 6 Research Associate, Department of Ichthyology, California Academy of Sciences, San Francisco, California, United States of America; University of Glasgow, United Kingdom

## Abstract

Detailed descriptions of morphological features, morphometrics, neurocranium anatomy, clasper structure and egg case descriptions are provided for the thickbody skate *Amblyraja frerichsi*; a rare, deep-water species from Chile, Argentina and Falkland Islands. The species diagnosis is complemented from new observations and aspects such as colour, size and distribution are described. Geographic and bathymetric distributional ranges are discussed as relevant features of this taxońs biology. Additionally, the conservation status is assessed including bycatch records from Chilean fisheries.

## Introduction

The genus *Amblyraja* Malm 1877 (Rajiformes: Rajidae) is a group of wide-ranging, circumglobal, stout bodied skates that are found mostly at higher latitudes [1] and in deep tropical waters [2]; mostly on outer continental shelves, insular shelves and slopes, and around seamounts [3]. The genus as currently includes ten nominal species with perhaps two additional undescribed species. In the south-east Pacific Ocean three poorly known species are recognized [4]: *Amblyraja doellojuradoi* (Pozzi 1935), *A. frerichsi* (Krefft 1968) and *A. georgiana* (Norman 1938).

*Amblyraja doellojuradoi* occurs from off Argentina and the Falkland Islands through the Magellan Strait to off Punta Arenas, Chile at a reported depth range of 51 to 642 m [5]. *Amblyraja georgiana*, a morphologically similar species to *A. frerichsi*, is an Antarctic species known from the Antarctic Peninsula [6], [7] and South Georgia Island from a depth range of 150 to about 800 m. Perhaps the least known species in the region is the thickbody skate *A. frerichsi*. Originally described from a type series of 35 subadult and immature specimens [8] collected between 800 and 1000 m off the Rio de la Plata, Argentina/Uruguay common fishing zone; this species has been reported as a rare species in deepwater off Brazil, Argentina and the Falkland Islands [9]–[11]. Recently, high bycatch rates of *A*. *frerichsi* have been reported from the Magellan Strait [12]. However, due to a lack of adult descriptive material and proper field guides, this species largely went unnoticed in Chile and Argentina deep-sea fisheries. The absence of comparative material and a species-specific description of adults have lead to unregulated bycatch landings and a lack of capture records due to misidentification [13], due in part, to a relatively high degree of variability of morphological characters for this family of elasmobranchs [2], [3].

Complementary descriptions of the anatomy are usually made to to clarify the taxonomic status of an important fishery species, which enabled on-board observers to determine species-specific landings [14]. Skates worldwide are taken in considerable numbers either as a directed fishery or indirectly as bycatch [15] and given that many of skates are landed in large numbers and in a mixture of species, it is critically important that accurate morphological descriptions of both adult and subadult fish are available.

The aim of this research is to provide a detailed morphological description of *A. frerichsi* from specimens taken as bycatch in Chilean waters. The neurocranium, clasper, dermal denticles and egg case morphology are presented as characters to facilitate identification among other *Amblyraja* species in the area. New information on the habitat and fisheries, together with a discussion of the related conservation implications for this species for the south-east Pacific are also provided.

## Materials and Methods

**Figure 1:**
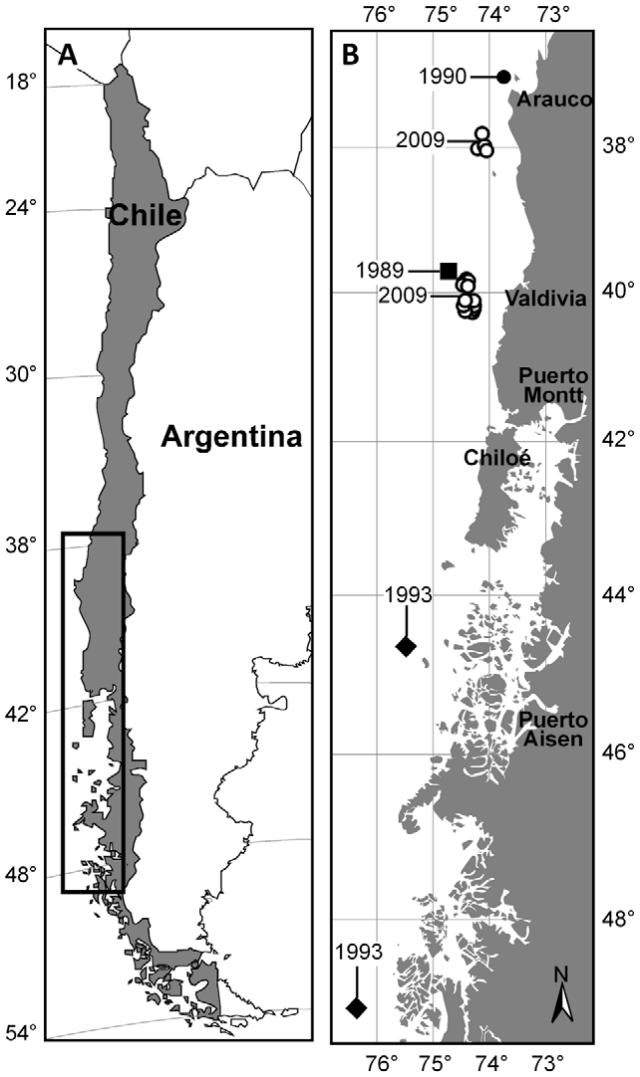
Map of Chile. (A) showing location of study area (inset box), and collecting localities (B) by date.

The specimens used in this study came from historical and recent bycatch records in the Patagonian toothfish (*Dissostichus eleginoides* Smitt 1898) fishery from southern Chile (Figure 1). A total of 13 males with sizes between 705 to 1201 mm total length (TL) (mean and standard deviation; 1068±163 mm), were caught between 1989 and 1993, and donated by artisanal fishermen to ELASMOLAB: two specimens were from off Valdivia (1989), four specimens were from Arauco (1990), and seven specimens were captured between Isla Guamblin and Golfo Ladrilleros (1993). In addition, 26 females ranging in size from 442 to 1762 mm TL (905±368 mm) and 26 males ranging between 404 and 1114 mm TL (835± mm) were caught in the same fishery during 2009 within the research project FIP No. 2008–60, five from off Arauco and 47 from off Valdivia, and for which sample collection permits were obtained from the Maritime and Fisheries Authority.

Morphometric measurements and description of key characters were made in 35 specimens. All measurements were made to the nearest millimeter (mm), and follow recent recommendations in the literature [16]. Terminology was adapted from recent literature for dermal denticles [17], egg capsules [18]–[19], and clasper [20]–[21] descriptions. The right clasper was removed from two adult males (1194 mm and 1201 mm TL) and both external and internal structures are described. The neurocranium was dissected and described from an adult male (1194 mm TL).

General biological data were also recorded to estimate the size at maturity, females were considered mature when yellow ovarian follicles and/or egg capsules were present [22]. Males were considered mature if sperm were present in their seminal vesicles, and their claspers were calcified and rigid [23].

Two adult specimens, one male (1094 mm LT) and one female (1213 mm LT, were preserved and deposited in the Marine Fishes Collection of Universidad Austral de Chile (Museum accession number IZUA-PM 4064 and IZUA-PM 4065). Egg capsules were extracted from a 1445 mm LT female and deposited at Universidad de Valparaiso Marine Fishes Collection (Museum accession number CCM-173 and CCM-174).

## Results

*Amblyraja frerichsi* (Krefft 1968).

**Figure 2:**
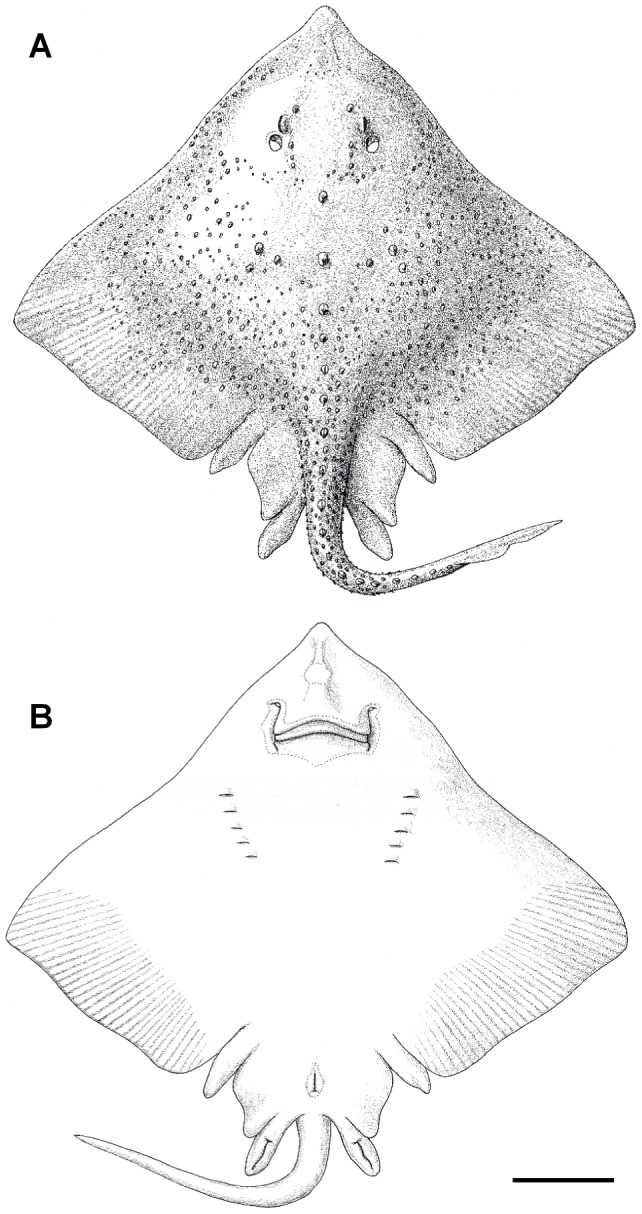
Adult male of *Amblyraja frerichsi* (1051 mm TL) in dorsal (A) and ventral (B) views. Scale bar 100 mm.

**Figure 3:**
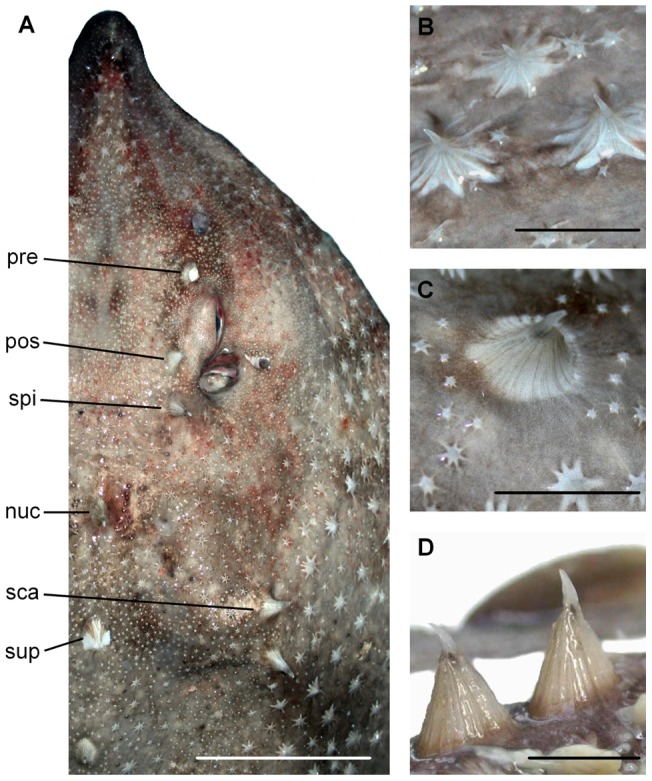
Dermal denticles of *Amblyraja frerichsi*. Dorsal view of the head (**A**) showing the snout and orbito-spiracular, nuchal and scapular thorns. Detailed view of malar (**B**), midline (**C**), and tail (**D**) thorns. (pre) preorbital, (pos) postorbital, (spi) spiracular, (nuc) nuchal, (sca) scapular, (sup) suprascapular. Scale bar 50 mm (**A**) or 10 mm (**B−D**).

**Figure 4:**
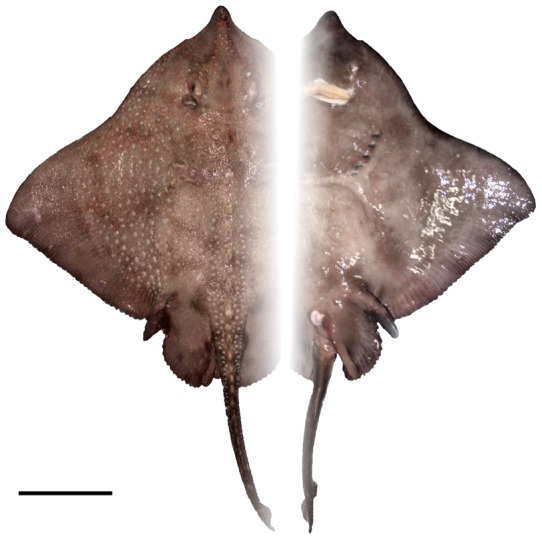
Coloration of *Amblyraja frerichsi* subadult male (985 mm TL) in dorsal (A) and ventral (B) views. Scale bar 100 mm.

**Figure 5:**
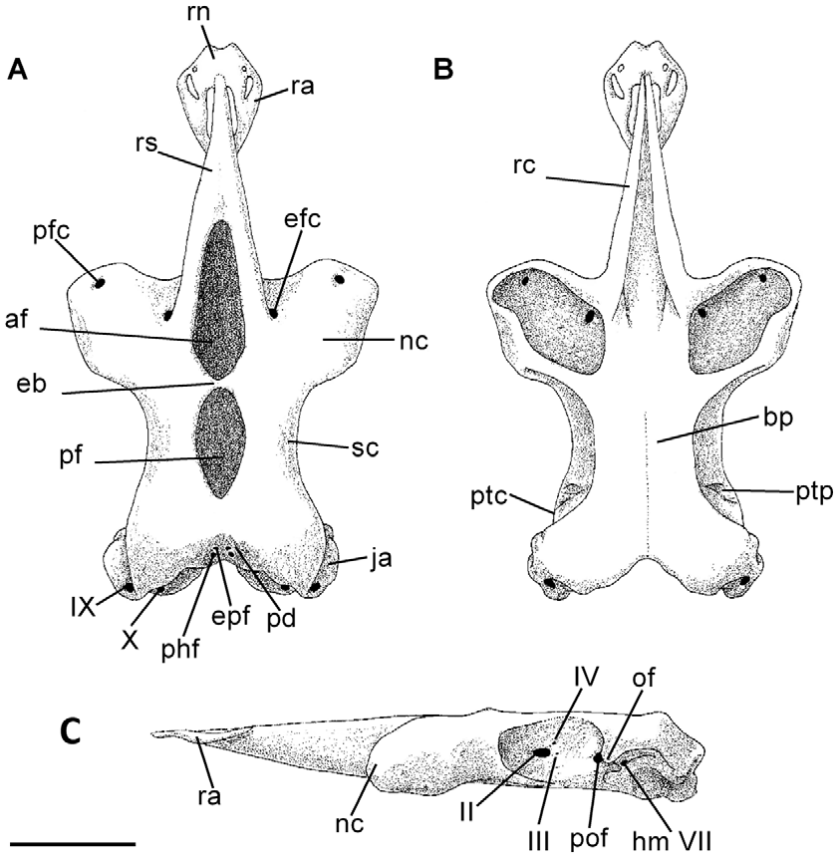
Neurocranium of *Amblyraja frerichsi* (adult male 1194 mm TL), in dorsal (A), ventral (B), and lateral (C) views. (af) anterior fontanelle, (bp) basal plate, (eb) epiphyseal bridge, (ecf) ethmoidal canal foramen, (ephf) endolymphatic foramen, (hm VII) hyomandibular branch foramen, (ja) jugal arches, (nc) nasal capsules, (of) orbital fissure, (pcf) preorbital canal foramen, (pd) parietal depression, (pf) posterior fontanelle, (phf) perilymphatic foramen, (pof) prootic foramen, (ptc) pteroptic crest, (ptp) pteroptic process, (ra) rostral appendix, (rc) rostral cartilage, (rn) rostral node, (rs) rostral shaft, (sc) supraorbital crest, (II) optic nerve foramen, (III) oculomotor nerve foramen, (IV) trochlear nerve foramen, (IX) glossopharyngeal nerve foramen, (X) vagus nerve foramen. Scale bar 50 mm.

**Figure 6:**
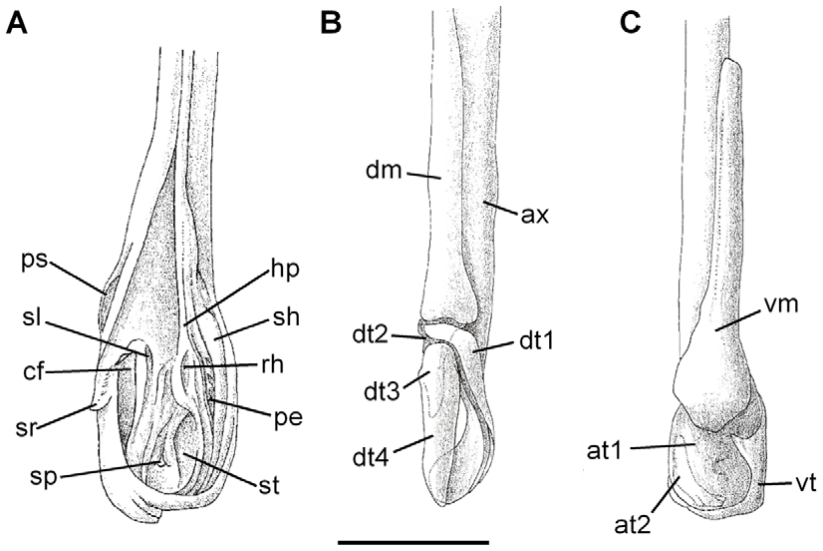
General structure of right clasper gland and cartilages of *Amblyraja frerichsi* in lateral. (A) view partially expanded; clasper cartilages in dorsal (B) and ventral (C) views. (at1) accessory terminal 1, (at2) accessory terminal 2, (ax) axial, (cf) cleft, (dm) dorsal marginal, (dt1) dorsal terminal 1, (dt2) dorsal terminal 2, (dt3) dorsal terminal 3, (dt4) dorsal terminal 4, (hp) hypopyle, (pe) pent, (ps) pseudosiphon, (rh) rhipidion, (sh) shield, (sl) slit, (sp) spike, (sr) spur. (st) sentinel, (vm) ventral marginal, (vt) ventral terminal. Scale bar 50 mm.

**Figure 7:**
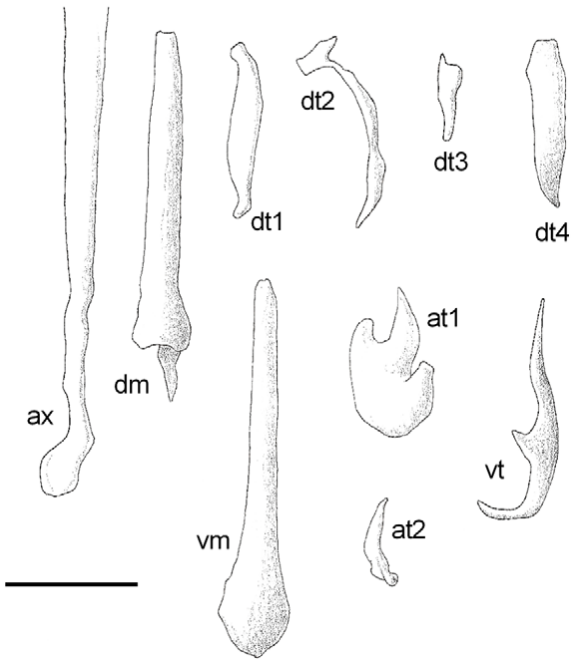
Dorsal view of clasper cartilages of *Amblyraja frerichsi*. Abbreviations of clasper cartilages are indicated at Figure 6. Scale bar 20 mm.

**Figure 8:**
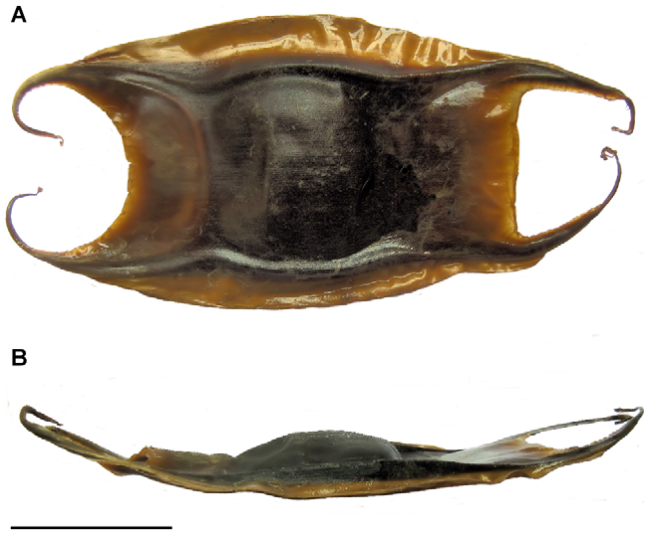
Egg capsules of *Amblyraja frerichsi* in dorsal (A) and lateral (B) views. Scale bar 50 mm.

Thickbody skate (Figures 2, 3, 4, 5, 6, 7, and 8; Tables 1, 2, 3, and 4).

**Table 1:** *Amblyraja frerichsi* catches location, sex and depth by year of historical and recent collections.

Year	Depth (m)	Sex	Geographical position
1993	1,900	4 Male	44°40′S; 75°30′W
1993	2,200	3 Male	49°01′S; 76°20′W
1989	1,300	2 Male	39°49′S; 74°24′W
1990	1,990	4 Male	37°00′S; 73°45′W
2008	2,200	3 Female	37°57′S; 74°12′W
2008	2,200	1 Female	38°01′S; 74°14′W
2008	2,200	1 Female	37°48′S; 74°12′W
2009	1,280	1 Male	40°08′S; 74°17′W
2009	1,550	1 Female	40°07′S; 74°23′W
2009	1,520	1 Male	40°05′S; 74°22′W
2009	1,253	3 Male; 3 Female	40°06′S; 74°23′W
2009	1,150	6 Male; 5 Female	40°02′S; 74°24′W
2009	1,445	1 Male	40°06′S; 74°18′W
2009	1,317	2 Female	40°06′S; 74°17′W
2009	1,614	3 Male; 3 Female	40°06′S; 74°26′W
2009	1,075	1 Male	40°06′S; 74°23′W
2009	1,037	1 Female	40°10′S; 74°18′W
2009	1,414	1 Male; 2 Female	40°08′S; 74°24′W
2009	1,419	1 Male	40°07′S; 74°26′W
2009	1,729	5 Male: 1 Female	40°05′S; 74°23′W
2009	1,168	1 Male	39°52′S; 74°26′W
2009	1,168	1 Male; 1 Female	39°52′S; 74°26′W
2009	1,168	1 Male; 1 Female	39°50′S; 74°22′W
2009	1,168	1 Female	39°53′S; 74°22′W

**Table 2:** Measurements (mm) of the morphometric characters taken from 35 specimens of *Amblyraja frerichsi*.

Morphometric character	Min.	Max.	Mean	s.d.
Disc width (DW)	70.77	80.74	77.39	2.54
Disc length (DL)	55.42	59.08	56.73	1.04
Snout length (preorbital direct)	12	14.78	13.46	0.91
Snout to spiracle	13	15.61	14.32	0.87
Head (dorsal length)	18.37	21.84	19.90	1.02
Eye diameter	2.47	3.16	2.87	0.22
Orbit diameter	2.91	5.07	4.35	0.57
Orbit and spiracle length	4.63	6.38	5.29	0.49
Spiracle length (main aperture)	2.57	3.27	2.81	0.23
Distance between orbits	6.47	8.04	7.10	0.40
Distance between spiracles	5.83	10.69	8.92	1.20
Snout to cloaca (1st hemal spine)	50.77	54.96	53.41	1.24
Distance-cloaca to caudal-fin tip	38.55	43.85	41.41	1.56
Ventral snout length (pre upper jaw)	10.59	15.49	12.66	1.44
Prenasal length	9.61	12.16	10.88	0.75
Ventral head length (to fifth gill)	26.3	30.81	29.26	1.29
Mouth width	9.66	12.33	11.23	0.74
Distance between nostrils	8.87	10.56	9.79	0.57
Nasal curtain (total width)	12.8	14.64	13.77	0.64
Width of first gill opening	1.48	2.16	1.76	0.22
Width of fifth gill opening	1.2	2.2	1.63	0.27
Distance between first gill openings	19.4	23.14	21.16	1.17
Distance between fifth gill openings	13.66	15.78	14.59	0.74
Length of anterior pelvic lobe	8.34	11.89	9.92	1.07
Tail at axil of pelvic fins (width)	3.22	4.65	3.88	0.38
Tail at first dorsal-fin origin (width)	2.5	3.36	2.94	0.28
D1 base length	3.63	4.87	4.35	0.43
D2 base length	3.53	5.69	4.89	0.63
D1 height	1.7	2.44	1.97	0.21
D2 height	1.57	2.13	1.86	0.20
Interdorsal space	0	1.77	0.60	0.56
Caudal-fin length	1.75	3.87	3.15	0.61
Tail lateral folding	35.52	41.93	38.31	1.89

Range expressed as percentage of total length (TL). Mean value (Mean) and standard deviation (s.d.) are indicated in each case.

**Table 3:** Measurements (mm) of the neurocranium taken from one specimen of *Amblyraja frerichsi* (adult male 1194 mm TL) expressed as percentage of nasobasal length.

	mm	%
Nasobasal length	128.9	100
Cranial length	225	174.6
Rostral cartilage length	112.5	87.3
Prefontanelle length	71.6	55.5
Cranial width	129.4	100.4
Interorbital width	58.35	45.3
Rostral base	40.4	31.3
Anterior fontanelle length	63.5	49.3
Posterior fontanelle length	20.7	16.1
Anterior fontanelle width	43.2	33.5
Posterior fontanelle width	20	15.5
Rostral cleft length	94.2	73.1
Rostral appendix length	44.8	34.8
Rostral appendix width	32.7	25.4
Posterior foramen of rostral appendix length	33.15	25.7
Cranial height	32.8	25.4
Rostral cartilage height	24.2	18.8
Width across otic capsules	90.5	70.2
Least width of basal plate	38.25	29.7
Greatest width of nasal capsule	50.65	42.2
Internasal width	33.5	26.0

**Table 4:** Measurements (mm) of the egg capsules of *Amblyraja frerichsi*.

	CCM-173	CCM-174
Central capsule length	115.5	112.3
Central capsule width	88.5	90.5
Capsule height	15	12.6
Anterior apron length	22.3	17.9
Right anterior horn length	59	59
Left anterior horn length	59	56
Posterior apron length	21.8	20.5
Right posterior outer horn length	54	59
Left posterior outer horn length	53.8	53
Right lateral keel	9.9	10.3
Left lateral keel	13.4	11.6

Synonymy: *Raja frerichsi* [6], [8], [24], *Raja* (*Amblyraja*) frerichsi [21], *Amblyraja frerichsi* [5], .

### Diagnosis

A relatively large species of *Amblyraja* with the following combination of characters: quadrangular disc, 1.3 times its length and width 77% TL; tail evenly tapering and robust, width at base 1.3 times first dorsal-fin origin; snout length 23% disc length. Ventral and dorsal disc uniformly dark-brown to grey-brown colouration. Dorsal surface densely cover with small dermal denticles on the head, fins and tail; large hook-shaped denticles on the orbital series, one preorbital, one postorbital and one postspiracular; also one nuchal, one scapular and three suprascapular are present in the scapular series; and 8−26 in the midline of disc, from behind the scapula to tail. Adult clasper relatively large, 49% caudal length; and distal lobe extremely spatulate. Violin-shaped neurocranium with large and bulbous nasal capsules, with a relatively large rostral shaft ending in a widened rhomboidal rostral node. Dark brown and finely striated egg capsules with posterior horns longer than the anterior, both tapering towards the tips. Horns of egg capsules without lateral fibrils and coiled terminal tendrils.

### Description

Disc quadrangular, 1.28 times as wide as long in adult specimens, width 77.4% TL, maximum disc width 59.1% TL (Figure 2). Anterior margin of disc concave behind head, convex at eye level and concave at spiracle level. Outer margin of disc is acute angled and posterior margin is slightly convex almost straight. Snout short, preorbital snout length 0.9 times snout to spiracle length, 1.9 times interorbital space, 23.7% disc length (DL). Snout tip pointed, lacking distal process or filament. Orbit length 2.5 times interorbital space. Spiracle small, 0.9 times orbit length, 4.9% DL; opening subcircular in shape. Nostril sub-triangular to oval; anterior nasal flap forms an opening, posterior lobes not developed meeting medially to form nasal curtain; distal end sub-rectangular with curved fringe on the posterior margin. Internarial distance 2.2 times distance between first gill slits, 1.5 times distance between fifth gill slits. Mouth slightly arched in subadult males and females, curved in adult males; mouth width 1.2 times internarial distance. Teeth with a flat oval base and acute single cusp, arranged in quincunx without sexual dimorphism. Total tooth count in upper jaw 36 (42) arranged in four rows and in lower jaw 37 (40) arranged in five rows. Bilobed pelvic fins thin and tapering toward distal end; anterior lobe length 5.7% DL, with a broad posterior lobe with concave external margin. Strong tail broad, depressed, tapers from broad base to first dorsal fin. Tail moderately long, length 43.3% TL; width at insertions of pelvic fins 1.3 times width at first dorsal-fin origin. Lateral tail fold long and well developed, length 38% TL, origin after dorsal fins and extending to tail tip. Dorsal fins strongly acute with long bases and similar shape. First dorsal fin base length 2.2 times height; second dorsal fin base length 2.6 times height. Both fin apices angular; straight posterior inner margins and anterior margins convex. Dorsal fins sometimes separated by a space, with interdorsal distance 13% first dorsal-fin base length. Second dorsal fin continues to a short, undeveloped epichordal caudal lobe, 0.7 first dorsal-fin base length. Main morphometric characters expressed as a percentage of total length are found in Table 2.

### Dermal denticles

Dorsal surface with a wide variety of dermal denticles, thornlets and thorns, but ventral surface with no squamation (Figure 3A). Small dermal denticles (myrmecoid type), less than 2 mm in height on subadults and adults, have radial symmetry and cover the surface of eyelids, pelvic fins and tail, including dorsal and caudal fins. Basal plate (BP) edge serration of dermal denticles resembles a 4−6 ridged star (Figure 3B). Thornlets are dermal denticles usually more than 3 mm height (in subadults and adults), similar in shape but larger and more robust than dermal denticles. Thornlets have a small BP with 7−9 straight furrows and elongated ridges; BP with a saddle-like fold and crown curved backward staying within the BP. Thornlets are densely distributed in rostral and malar zones beside pectoral fins and parallel, lateral rows in tail. Additionally, larger denticles that double in size and height thornlets (usually 60−80 mm height in subadults and adults) are called thorns, and are distributed around head (Figure 3C) and tail (Figure 3D). The orbital series has three thorns, two in the inner orbit proximal (preorbital) and distal (postorbital) margins, one behind spiracle (postspiracular); suprascapular series has one nuchal, one scapular and three suprascapular thorns, the latter forming a triangle. In the midline 18−26 thorns are present from the disc, behind the scapula at level of the maximum disc width, to the tail. Thorns BP have fine radial ridges; wavy-lined lobed edges in a regular elliptic shape without elongated ridges or furrows. Thorns acute and curved crown is only one third of BP height and reaches BP margin. Alar hooks (thorns-like) found only in adult males in six to eight longitudinal rows near the edge of pectoral fins. When an interdorsal space is present, one or two thornlets of variable size present.

### Colour

Dorsal disc of fresh adult and subadult male is dark-brown except blackish-brown distal edges, pelvic fins, tail, dorsal and caudal fins. Ventral surface uniformly dark-brown (Figure 4). Dorsal colouration of female is greyish-brown; disc margins, scapulae and pelvic fins darker. Tail, dorsal and caudal fins are blackish-brown. In adult males, dorsal and ventral surface is grey-brown with disc margins, scapulae, pelvic fins, tail and snout a darker shade. Anterior lobe of pelvic fins, mouth edges, nasal curtain, cloaca and gill slits are white in all individuals. Sensory pores clearly visible on the ventral side around the snout and mouth margins. Sometimes there is a white triangular patch, where the first two vertices point to the fifth gill slit and the third vertex to the cloaca. Another oval spot can be seen usually at the end of the nose. Immature specimen (<400 mm TL) may have white blotches between gill slits.

### Neurocranium

Neurocranium is violin-shaped (Figure 5). Nasal capsules (**nc**) are large and bulbous, orientated 70° forward midline rostral cartilage. Ethmoidal canal foramen (**ecf**) is located beneath rostral base, between rostral cartilage (**rc**) and nasal capsules (**nc**). Preorbital canal foramen (**pcf**) is located in the distal margin of nasal capsules in the same longitudinal line of the jugal arches (**ja**). Strong, angular jugal arches surrounding the parietal fossa (**pfs**), with perilymphatic (**phf**) and endolymphatic (**eph**) foramina located in the inner margins. Rostral node (**rn**) is rhomboidal in shape with a pronounced concavity at the anterior margin. Rostral appendices (**ra**) have two small foramina, the larger with an inverted triangular-shape. Rostral appendices are *c*. 37% of rostral shaft (**rs**) length. Fontanelles separated by a small epiphyseal bridge (**eb**). Anterior fontanelle (**af**) is oval with a high resemblance to a blunt arrowhead; while posterior fontanelle (**pf**) is ovoid. Posterior fontanelle *c*. 68% of anterior fontanel length. Openings of nasal capsules are kidney-shaped with larger length in same orientation of nasal capsule. The lateral cranial roof is dorsally limited by the supraorbital crest (**sc**), with no visible foramina on surface. Neurocranium basal plate (**bp**) is short and narrow at orbit level. Dorsolateral and delimiting the otic capsule, is located the rounded pterotic process (**ptp**) which is continuous towards a low pterotic crest (**ptc**). Optic nerve foramen (**II**) is elliptic and rounded, located in mid-orbital wall. Dorsal to the optic nerve foramen are two small openings for the passage of the trochlear nerve (**IV**). Posterioventral to the optic nerve foramen is the foramen for the oculomotor nerve (**III**). Prootic foramen (**pof**) is located in distal margin near orbital fissure (**of**) and hyomandibular branch of facial nerve foramen (**hm VII**). The remaining branches of the trigeminal and facial nerves (**V**+**VII**) cannot be observed at naked eye. Morphometric measurements, expressed in mm and percentage of nasobasal length are shown in Table 3.

### Clasper

Mature males of have moderately long, solid and distally widened claspers. Clasper length can reach 49% (36−50%) of caudal length in mature males, and all surfaces lack dermal denticles. In the dorsal surface (Figure 6A), the pseudosiphon (**ps**) is located in the distal half of clasper and is continued by the cleft (**cf**), a narrow longitudinal fissure at same line with the rhipidion (**rh**). The slit (**sl**), a tissue infolding made by the division of axial cartilage and the proximal end of dorsal terminal cartilage 2, is located in the proximal half of clasper between **ps** and **cf**. In the outer lateral margin of dorsal lobe and in same line with **rh**, the spur (**sr**) is formed by the distal end of dorsal cartilage terminal 3. The shield (**sh**) is an elongated plate located in the internal ventral lobe, dorsally convex with sharp edges extending from the hypopyle (**hp**) up to the pent (**pe**) and the sentinel (**st**) on its distal margin. The rhipidion (**rh**) is observed as a fan-shaped protruding structure which extends parallel to the clasper longitudinal axis and continues to **pe**, a structure deeply folded and covered with tissue. In its distal inner margin **st** is located, a thick structure, dorsally convex, funnel-like composed by the distal margin the terminal accessory cartilage 1. The spike (**sp**) is a small tab covered by tissue, with the tip pointing towards the dorsal lobe.

The clasper skeleton (Figure 6B) consists of the ellipsoidal axial cartilage (**ax**), that flattens dorso-ventrally towards distal margin where is spatulate; the **ax** is attached at two-thirds of its length to the dorsal marginal (**dm**) and ventral marginal (**vm**) cartilages. In the distal margin **ax** continues to the dorsal terminal cartilage 4 (**dt4**). Dorsal marginal cartilage (**dm**) is subrectangular, flattened and concave, tapering toward distal end and it expands abruptly to the joint to axial cartilage (Figure 6B−7). Dorsal terminal cartilage 1 (**dt1**) is long, curved and flattened, with sharp edges (Figure 7). The small curved t-shaped dorsal terminal cartilage 2 (**dt2**) is located at **dt1** proximal end; while the dorsal terminal cartilage 3 (**dt3**), is a flat triangle connected to **dt2** in the distal margin (Figure 6B−7). Long, spatulated dorsal terminal cartilage 4 (**dt4**) joins dt3 in the middle dorsal proximal end. Ventral marginal cartilage (**vm**) elongated with straight edges, ventrally curled and acute in the distal end (Figure 6C−7). Hook-shaped ventral terminal cartilage (**vt**) has a wide-base projection in middle of the inner edge. Distal half of **vt** surrounds accessory terminal cartilage 1 (**at1**) and is connected to **dtr1** distal end. Proximal half of **at1** has an acute, strongly curved appendix (Figure 7), located below the **vmg** and connecting to **ax**. Accessory terminal cartilage 2 (**at2**) is small, curved claw-like, located below **at1** while proximal end is located under inner edge of **vm** and **at1** junction (Figure 7).

### Egg capsules

Capsules *in uteri* were non-translucent, dorsoventrally flattened and thick walled. Dark brown coloration in the centre of the capsule faded to light-brown in the outer margins (Figure 8A). Surface is finely and longitudinal striated, giving a smooth surface texture. These striations, hard to distinguish to the naked eye, are present on the entire capsule surface including horns. In a lateral view, the dorsal face is convex, while the ventral face is flattened (Figure 8B). Anterior and posterior margins of the egg capsules are secured by a thick apron while lateral borders exhibited strong lateral keels. Anterior apron is concave and against transmitted light, only the last *c.* 7 mm of the anterior margin looks translucent. Posterior apron have a straight posterior margin, which last *c.* 10 mm is translucent. Thick and strong lateral keels were also present the almost full length of the capsule, extending onto the base of anterior and posterior horns, wider in the middle of the egg capsules and thinner towards both anterior and posterior margins. Outer margins of lateral keels are slightly rounded giving the egg capsules a typical barrel shape. Egg capsules do not have any lateral or any other accessory adhesion fibres. Anterior horns are thick, dorsoventrally flattened and inwardly curved thought not long enough for crossing over. The inner margin of the horn is united to the anterior apron. The tips of these horns are fibrous showing a very little coiled end. Posterior horns are straighter and longer than anterior, and also inwardly curved on the tips. The inner side of posterior horns are fused with the posterior apron and became thinner with a tendril-like final portion. Nevertheless, only the tips are thin and like the anterior horns, posterior horns have the same terminal entanglement. Measurements for egg capsules are presented in Table 4.

### Size

Specimens examined range in size from 404 to 1762 mm TL. No individuals with umbilical scars were observed but based on the egg capsules’ maximum size and the smallest skate specimens recorded, the size at birth seems to be about 216 mm TL. Immature specimens range from 216 to 817 mm TL [8]. Adult females (mature or egg-bearing) ranged from 965 to 1731 mm TL. Males over 855 mm TL had fully calcified claspers suggesting maturity is attained between 775 and 907 mm LT. No apparent sexual dimorphism were observed in size and disc shape.

### Distribution

Known distribution on both sides of southern South America, from Rio de Janeiro, Brazil (22°S) through the Magellan Strait to Arauco, Chile (37°S). Recorded at depths of 600 to 2,609 m in the Atlantic Ocean [1],[8]–[9] and 1,037 to 2,200 m in the Pacific Ocean.

## Discussion

The geographical range of *Amblyraja frerichsi*, based on verified records in the literature, extends southwards from Rio de la Plata (35°S) [8] to the Falkland Islands [10] and Cape Horn (57.3°S) [12] in the south-west Atlantic; and northwards in the south-east Pacific, from Cape Horn through the Patagonian Archipelago to 52°S [12]. Our observations extend the latitudinal range in the south-east Pacific to Arauco (37°S); about 1,700 km north of the previous confirmed records. It should be noted, however, unverified records of single specimens have been reported from Coquimbo (30°S) [27] and the Tarapacá Region (23°S) [28] in the south-east Pacific and Rio de Janeiro, Brazil (22°S) in the south-west Atlantic. These isolate records may indicate a more extensive northerly range on each side of South America than is currently accepted.

No major differences in the anatomy were found when compared to the original description [8]. However, differences were found in the spinulation pattern with 16–26 midline thorns found in comparison to the original description (16–22 thorns [8]). Colour pattern was also similar to that provided in the original description, although this character is sensitive to environmental influences [29] and colour in known to vary in this genus with immature individuals often darker than adults [30]. No ontogenetic growth differences were observed in size and disc shape between sex and maturity stage.

Internal and external morphology of claspers may help to define the genus assignment in species specific complex [20], [21], [31] as has been documented for deep-water skates, *i*.*e*. the *Zearaja* and *Dipturus* complex [32]. Recently, a comprehensive study of the clasper in three *Amblyraja* species have been published [33] contributing to enhance the species diagnosis as a major component of genus morphology.

Structural consistencies can be detected in the clasper internal cartilages within *A*. *frerichsi* and *A. doellojuradoi, A*. *hyperborea* (Collett 1879) and *A*. *radiata* (Donovan 1808) [21], ; especially in the shape of the dorsal terminal 2 (**dt2**) and 4 (**dt4**); the ventral terminal and the accessory terminal 1 (**at1**) and 2 (**at2**) cartilages. But the shape and disposition of dorsal terminal 1 (**dt1**) and 2 (**dt2**) cartilages are slightly different; and these differences together may be used in the species diagnosis.

As seen in other skates, the reproductive mode of *A. frerichsi* is single oviparity, in which two egg capsules are produced simultaneously, one in each nidamental gland. Previous descriptions of the egg capsules of *Amblyraja* species are restricted to a few species: egg capsules of *A. hyperborea* and *A. robertsi* (Hulley 1970) are bigger than the mean size of those described in this study [35], [36]; in contrast to the egg capsules of *A. radiata* that appear to be smaller [37]. Nevertheless, all share some common features, such as colouration pattern, texture, apron thickness and lateral keels. The absence of accessory lateral adhesion fibres and long non tendril-like horns are features shared with *A*. *robertsi* and *A. hyperborea*. Egg capsules of *A. radiata* have a dense and entangled bundle of fine adhesion fibres at the top of each anterior horn, which makes them longer than the posterior horns. In all *Amblyraja* egg cases, the posterior horns are longer than the anterior ones, and taper towards their tips. The general absence of lateral fibrils or long terminal tendrils suggest that *Amblyraja* species deposit egg capsules on the sea floor, rather than attach them to debris or any other types of substrata.

*Amblyraja frerichsi* could be potentially confused with other *Amblyraja* species present south-east Pacific Ocean because of similarities in the distribution patterns of dermal denticles and its general morphology. However, it can be distinguished from *A*. *doellojuradoi* who have white ventral colouration and 12–15 midline thorns [11], [38]. Also it can be differentiated from *A*. *georgiana*, who have large white blotches on the ventral surface and 20–28 midline thorns [10], [38].

An important aspect of this taxon is the geographical and bathymetric range expansion in the Chilean coast. Capture records in south Chile (southwards of 40°S) were made at shallower depths (800−1,300 m) than in northern locations (1,900−2,200 m). These depth variations could be evidence of a relationship between latitudinal ranges, catch depths and water mass temperature; a hypothesis proposed for *Rajella nigerrima* (De Buen 1960) [39] on the continental slope of Chile. The environmental homogeneity due the Antarctic Intermediate Water mass that flows at depths of 700 to 1200 m along the Chilean continental slope [40], may explain the depth range variation along the latitudinal gradient in the south-east Pacific.

In the south-west Atlantic, capture records of *Amblyraja frerichsi* are scarce and restricted to deep waters outside the continental shelf between 600 and 1,000 m [5], [9]. In the same area, off Rio de la Plata, only immature specimens have been captured predicting that adults would be at greater depths [8]. In Chile, *A*. *frerichsi* specimens were captured over the continental slope at depths between 800 and 2,200 m. All female captured shallower than 1,300 m were immature. Sexually mature female were caught at greatest depths while mature males were observed over the whole depth range. Such depth-segregation by maturity occurs in a number of elasmobranch species [41], [42]. Spatial and sexual segregation in *A*. *frerichsi* could be related to reproductive events, such as oviposition [43], [44]. Chilean egg-bearing females were caught in deeper zones as well as deep-water corals (genus *Antipathes* and *Bathypathes*), showing a positive correlation in the bycatch. Similarly, in the area is thought that the dusky catshark *Bythaelurus canescens* (Günther 1878) use deep-water coral branches as a substrate for egg-laying [45]. But as other *Amblyraja* species, *A*. *frerichsi* may deposits its egg capsules on the sea bed and potentially use the coral gardens as oviposition shelter zones. Tendrils and curved horns are usually associated with oviposition into complex three-dimensional substrates, whereas egg cases without tendrils are generally deposited on bare substrate [45], [46]. The reason for female's apparent preference for deeper water is uncertain, but mature female could have a male-avoidance strategy [47] going deeper to lay her eggs, with young skates migrating to relatively shallow waters to reduce intraspecific competition [48]. Sex-specific habitat use has been reported previously for batoid species [42], [43].

Sexual segregation may compromise the integrity of the population if only mature or immature fraction is caught or incidentally harvested [49]. A high abundance and bycatch interaction of *A*. *frerichsi* have been reported [12] in the *D. eleginoides* fishery around the Patagonian coast of the south-east Pacific Ocean (52°S to 57°S). In this area, *A*. *frerichsi* represents 97% of the elasmobranch bycatch by species and 57% of the elasmobranch biomass caught in this fishery. In the same fishery but offshore from Valdivia (40°S), have been reported a low interaction/abundance of *A*. *frerichsi*, comprising just 1% of the total catch [13]. In this fishery while only 8% of the hauls were made below 1,200 m (between 1,200 and 1,800 m), 65% of the *A*. *frerichsi* catch comprised mature females. The high effort and pressure made by the fleets in the Patagonian greatly exceeds the few artisanal vessels that continue catching *D*. *eleginoides* [50]; therefore, more immature skates are caught and discarded than mature fraction maintaining an ephemeral population balance until bycatch will be evaluated over the entire fisheries distribution range. The relatively higher abundance in the southern records, regarding the capture depth range extension, supports the observation that *A*. *frerichsi* prefers cold waters, as do almost all species of this genus, except *A*. *reversa* (Lloyd 1906) who inhabits deepwater of Arabian Sea [51].

The present study extends the distributional range in the south Pacific Ocean from 36°S to the Patagonian Channels (54°S), with a continuous presence along the lower continental slope of Chile. Besides this geographical extension, the bathymetric distributional range is also extended, inhabiting between 800 and 2,200 m depth. It is urgent to focus research in *A. frerichsi* population sex and maturity structure, especially in the area where it occurs as bycatch in the Patagonian toothfish (*D*. *eleginoides*) fishery from the southern channels of Chile, where overfishing could threaten the integrity and balance of this skate's population.
